# The race against the "septic shark"

**DOI:** 10.1186/cc14729

**Published:** 2015-12-18

**Authors:** Martin Westphal, Tim Kampmeier

**Affiliations:** 1 Department of Anesthesiology, Intensive Care and Pain Medicine, University Hospital of Münster, Albert-Schweitzer-Campus 1, Bld. A1, D-48149 Münster, Germany; 2 Fresenius Kabi AG, Else-Kröner-Str. 1, 61352 Bad Homburg, Germany

## Abstract

Great white sharks are responsible for about 10 cases of death annually worldwide, as compared with millions of deaths caused by sepsis. However, the basic principles of avoiding shark attacks and fighting sepsis seem to be similar: avoidance, attention, and speed, if necessary. The present review discusses the current status of the systemic inflammatory response syndrome (SIRS) criteria, which are actually content for discussion because of their low specificity. Current data suggest that one in eight patients with severe sepsis does not fulfill the SIRS criteria and is consequently missed, and therefore the calls for new definitions of sepsis are getting louder. Furthermore, the need for early treatment of sepsis and fast admission to an intensive care unit (ICU) with experienced stuff is reviewed as well as the early and appropriate initiation of therapy, namely antibiotic and volume therapy. A key feature is the analysis of the studies from the so-called "Sepsis Trilogy" (ProCESS, ARISE, and ProMiSe studies), with a focus on the status of early goal-directed therapy (EGDT). The authors of the "Sepsis Trilogy" concluded that there is no benefit regarding survival in septic patients by using EGDT as compared with standard therapy. However, the low mortality of the control groups within the "Sepsis Trilogy" studies as compared with the Rivers et al. study from 2001 leads to the conclusion that there has been an improvement in the therapy of septic patients, most probably due to the early initiation of therapy as a kind of "standard" in sepsis therapy. Finally, the phenomenon of a "large trial disease" is discussed, exemplary in a trial which investigated the maintenance of the "right" mean arterial pressure in sepsis patients. Even if the result of a large randomized trial might be that there is no difference between two study groups, the real exercise is to identify the patient collectives who might benefit or experience harm due to an intervention. In summary, as compared with swimming in dangerous waters, high attention is needed in handling septic patients. Once an attack has occurred, speed is of utmost importance (i.e., initiation of therapy and admission to the ICU) because it appears logical that time is critical in septic patients This may have resulted in the implementation of early (goal-directed) treatment as a "standard" in the treatment of sepsis with significant improvement in survival.

## 

Now is no time to think of what you do not have. Think of what you can do with what there is.

Ernest Hemingway (*The Old Man and the Sea*, 1952)

Great white sharks are responsible for about 100 unprovoked attacks on human beings and 10 cases of death annually worldwide [1]. These animals are excellent and fast swimmers with a swim speed of about 40 miles/hour [2]. To minimize the risk of potentially deadly contact with a great white shark, one should address three basic principles: avoidance, attention, and speed, if necessary.

Sepsis and septic shock are responsible for millions of deaths every year [3]. Despite progress in improving outcome, the number of sepsis treatments has significantly increased in the last decade [4]. Interestingly, the key principles in fighting sepsis have some similarities with the principles of minimizing shark attacks; that is, prevention (avoidance), focus on the best treatment algorithms (attention), and timely correction of the underlying problem (speed).

Avoidance and prophylaxis of sepsis represent very important aspects to reduce the global burden of sepsis. In this context, awareness and education campaigns involving professional societies, the World Health Organization, and politicians that highlight the importance of hygiene and microbiology are very important measures.

After the consensus conference of the American College of Chest Physicians (ACCP) and the Society of Critical Care Medicine (SCCM) defined criteria for systemic inflammatory response syndrome (SIRS) and sepsis in 1992, these criteria have been used for diagnosis over more than two decades. Notably, Jean-Louis Vincent mentioned in 1997 [5] that the SIRS criteria, although highly sensitive, are of low specificity and therefore potentially misleading in making the right diagnosis. In this context, it was highlighted that two-thirds of ICU patients, as well as a high number of regular ward patients, actually meet the SIRS criteria. Importantly, these criteria neither take into account the underlying pathophysiology nor the severity of the inflammatory response [5].

Recently, Bellomo et al. [6] investigated the validity and sensitivity of the SIRS criteria in a retrospective analysis that included more than 100,000 patients over the last 14 years. The authors analyzed patients with infection and organ dysfunction and classified them as "SIRS-positive severe sepsis" (87.9%) and "SIRS-negative severe sepsis" (12.1%). Based upon this evaluation, it appears that approximately one in eight patients does not show the SIRS criteria even though they are suffering from septic shock. As expected, patients with "SIRS-negative severe sepsis" had a lower (but still relevant) mortality when compared with the "SIRS-positive" patients, underlining the risk of missing the right diagnosis in these patients [6]. This, in turn, indicates that the SIRS criteria in their current from are of limited use and may even be dangerous. Meanwhile, the calls are getting louder for the need for new definitions of SIRS and sepsis. In this regard, it has been proposed to better define sepsis as "a systemic response to infection with the presence of some degree of organ dysfunction" [7].

Another aspect of "attention" that goes along with early diagnosis of sepsis is the fast identification of the underlying problem, therefore prompting initiation of appropriate therapy. It appears to be logical that experienced intensive care physicians are more skilled in making the right diagnosis and initiating the best treatment of a septic shock as compared with physicians with little intensive care experience. This implies that patients suffering from a systemic inflammation may profit from an early (e.g., postoperative) admission to the ICU as compared with no or delayed admission. Furthermore, early ICU admission may help to prevent complications (due to disease progression), collateral damage, and costs. In this regard, it is important to note that several trials in critically ill patients provided evidence that an early intervention and admission to the ICU significantly improved patient outcome [8,9].

One option to improve patients' care might be the implementation of a "sepsis intervention team" (SIT), similar to a shock team in the emergency department. The SIT of a hospital could be a group of ICU-experienced physicians and special nurses with the mission to provide early diagnosis and initiate effective and adequate therapy for patients suffering from sepsis as fast as possible. As a hypothesis, early admission and therapy might also result in early discharge from the ICU, which could finally help to increase the overall ICU capacity. However, this assumption needs to be verified by future in-depth analyses.

A consequence of the early detection of sepsis and fast admission to the ICU should be timely initiation of the appropriate therapy. According to the current guidelines of the Surviving Sepsis Campaign (SSC) [10], effective antimicrobial therapy should be initiated within the first hour after diagnosis. The dimension of time in this context has been investigated by --among others--Kumar et al. [11], who reported an increase in mortality of 7.6% per hour delay in effective antibiotic therapy in hypotensive septic patients. Furthermore, the authors noticed in their retrospective study that only 50% of patients received effective antimicrobial therapy within the critical first 6 hours. In another study, Gaieski et al. [12] demonstrated a significant increase in mortality of septic shock patients, who received delayed appropriate antibiotic therapy more than 1 hour after triage. This implies that both time and the choice of the right antibiotic therapy are of utmost importance for patients suffering from septic shock. The SSC guidelines do not recommend special antibiotics, but the choice of an effective compound is recommended under consideration of all possible pathogens as well as their ability to penetrate tissue [10]. This all leads to the conclusion that the most important aspect concerning the antibiotic treatment of patients with septic shock is a fast initiation of the most effective and appropriate compound.

Supportive therapy represents another cornerstone in the fight against sepsis. In this regard, the choice of the "right" infusion solutions and vasopressor agents are of importance. The concept of early hemodynamic optimization is recommended by the SCCM guidelines [13]. This is, at least in part, related to the report of Rivers et al. [14] indicating significant improvement of survival in patients who received early goal-directed therapy (EGDT) as compared with conventional hemodynamic management. After this trial was published in 2001, EGDT became a "solid rock" in sepsis therapy for more than a decade. However, the concept of EGDT was heavily challenged after the Protocolized Care for Early Septic Shock (ProCESS) study was published in May 2014. The ProCESS Investigators randomized 1341 patients with sepsis into "protocol-based EGDT", "protocol-based standard care", and "usual care" [15]. Interestingly, there was no significant difference between groups regarding the primary endpoint; that is, 60-day mortality (protocol-based EGDT: 21%, protocol-based standard therapy: 18.2%, usual care: 18.9%; each *p *>0.3). Furthermore, the authors reported no significant differences in important secondary outcome variables such as need for organ support, 90-day mortality, and 1-year mortality [15].

A few months later, the Australian Resuscitation In Sepsis Evaluation (ARISE) study was published. This trial was designed to compare the effects of EGDT versus usual care in 1600 patients with early septic shock regarding all-cause mortality after 90 days [16]. Again, there were no significant differences among groups concerning 90-day mortality (EGDT: 18.6%, usual care: 18.8%; *p *= 0.90). Secondary and tertiary outcome parameters included hospital length of stay, need for and duration of organ support, 28-day and 60-day mortality, as well as adverse events. Patients allocated to the EGDT group were more likely to receive vasopressor agents (66.6% vs. 57.8%), blood transfusions (13.6% vs. 7.0%), and dobutamine (15.4% vs. 2.6%; each *p *<0.001) [16].

The third publication from this "sepsis trilogy" was the Protocolized Management in Sepsis (ProMiSe) study. This randomized controlled trial from the United Kingdom included 1260 patients with early septic shock and investigated the effects of EGDT vs. usual care on all-cause 90-day mortality. Secondary endpoints included the Sequential Organ Failure Assessment (SOFA) score at 6 and 72 hours, ICU length of stay, need for organ support, and quality of life [17]. Once again, there was no significant difference regarding 90-day mortality between the EGDT and the usual care groups (29.5% vs. 29.2%, relative risk = 1.01; *p *= 0.90). Concerning the secondary outcome measurements, patients randomized to the EGDT group had a significantly higher SOFA sore at 6 hours, received more advanced cardiovascular support, and had a longer length of stay in the ICU when compared with the usual care group (each *p *<0.05) [17].

Based upon the results of these studies, the authors concluded that protocolized EGDT did not improve outcome in patients suffering from sepsis [15-17]. However, this conclusion should be interpreted with caution to avoid a potentially dangerous misinterpretation. In this context, it is important to note that the mortality rates of the control groups in the ProCESS, ARISE, and ProMiSe studies were much lower as compared with the Rivers et al. study 13 years earlier, in which the control group experienced a hospital mortality of 46.5% [14] (Figure 1 and Table 1). So how can we explain a drop in mortality of 27.6% (or more) within 13 years? The most logical explanation is effective early intervention in the treatment of all sepsis patients over the last years, which most probably also affected the mortality in the control groups of the referenced trials [15-17].

**Figure 1:**
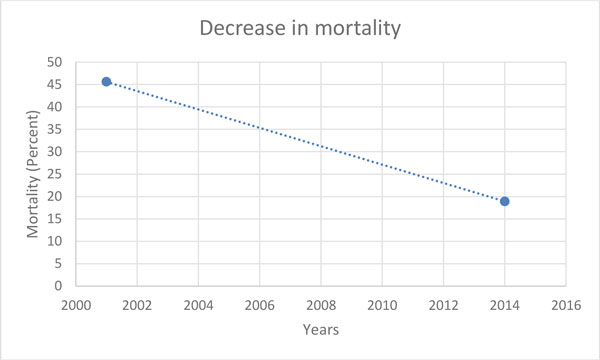
**Decrease in control group mortality from 2001 to 2014**. The decrease in mortality of the control groups from the 2001 Rivers et al. study [14] and the 2014 Australian Resuscitation In Sepsis Evaluation study [16].

**Table 1:** Mortality in EGDT and control groups (%)

	Mortality			
	
Study	In-hospital EGDT/control	28-day EGDT/control	60-day EGDT/control	90-day EGDT/control
Rivers et al. (2001) [14]	30.5/46.5	33.3/49.2	44.3/56.9	N/A
ProCESS (2014) [15]	N/A	N/A	21.0/18.9	31.9/33.7
ARISE (2014) [16]	N/A	14.8/14.9	N/A	18.6/18.8
ProMiSe (2015) [17]	N/A	24.8/24.5	N/A	29.5/29.2

Data presented as EGDT/control

The term "control" is used synonymously for "standard care" (Rivers et al.) and "usual care" (ProCESS, ARISE, and ProMiSe studies)

*ARISE *Australian Resuscitation In Sepsis Evaluation, *EGDT *early goal-directed therapy, *N/A *not available, *ProCESS *Protocolized Care for Early Septic Shock, *ProMiSe *Protocolized Management in Sepsis **{AU Query: Confirm table. You forgot to mention that the fisrt number represents EGDT and the second control as described in the original table}**

Time is critical from physiological, medical, and logical points of view. Early hemodynamic stabilization obviously became the new standard of care, which may have contributed to the significant reduction of mortality during the last decade. This could be interpreted as an important step forward in modern medicine. However, why are the mortality rates between the EGDT and control groups of the sepsis trilogy trials so similar (see Table 1)? In this regard, one may speculate that there is a threshold in supportive sepsis therapy and EGDT, where the effects of this concept are already maximized so that no further improvement may be achieved. Whether this assumption is true, or other concepts might provide further benefit for the septic patient, needs to be elucidated in future clinical trials.

Vincent and De Backer [18] nicely summarized the four phases in the treatment of shock: salvage, optimization, stabilization, and de-escalation (SOS-D). This concept, which has also been described by Hoste et al. in a slightly modified version, focuses on the clinical phases of shock from the initial "Salvage" or "Rescue" phase (where maintenance of a life-saving blood pressure with bolus infusion of fluids is recommended) until the "De-Escalation" phase (where a negative fluid balance should be achieved) [18,19]. Whether these concepts provide a benefit for the patient has likewise to be clarified. However, the idea to rapidly adapt fluid therapy to the respective pathophysiological phases of shock appears to be logical and may represent one of the crucial determinants in fighting septic shock (i.e., a timely and rational approach).

Another aspect of hemodynamic stabilization is the maintenance of the "right" mean arterial pressure (MAP). However, how do we know what the "right" MAP is? The current sepsis guidelines recommend a MAP of at least 65 mmHg within the first 6 hours [10]. Nevertheless, a "one size fits all" solution seems extremely unlikely with respect to the heterogeneity of the common patient collective. In this context, Asfar et al. performed a prospective clinical study investigating the effects of different MAP levels on mortality and organ dysfunction in patients suffering from septic shock. Seven hundred and seventy-six patients were randomized to receive therapy either with a MAP of 65-70 mmHg (low-target group) or 80-85 mmHg (high-target group) [20]. The authors noticed no difference between the groups regarding 28-day and 90-day mortality. However, the patients in the high-target group had a higher incidence of atrial fibrillation, and patients suffering from chronic hypertension in the low-target group received more renal replacement therapy [20]. Although the overall mortality among groups was similar, one should not conclude that the interventions were without any effects.

Perhaps we have to be more careful to interpret data of large trials and should unmask typical "large trial diseases". If no individual therapeutic targets are set and achieved in a study, the overall outcome is unlikely to be positive. The reason for this is that there may be patients in each treatment arm who experience benefit, neutral effects, or harm by the same intervention (Figure 2). As a consequence, the overall effect will be interpreted as not being different. It may thus be misleading to conclude that the intervention per se is useless. The aim should rather be to identify the subpopulations of patients who experience harm or benefit due to the intervention and to analyze why these effects occurred. This would provide much more information than the simple conclusion that there are no differences between the groups regarding overall long-term mortality.

**Figure 2:**
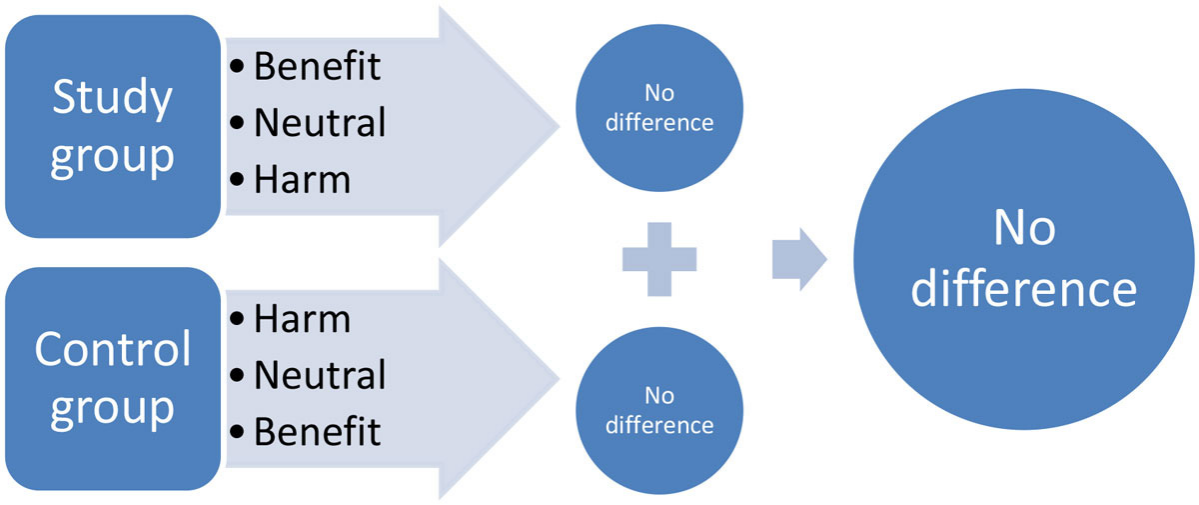
**"Large trial disease"**.

Taken together, the basic principles of fighting sepsis are similar to the rules of preventing shark attacks: once one is swimming in dangerous waters, high attention is needed. However, if one has bad luck and a shark approaches with the intention to attack, high speed may be important to survive. Similarly, time is critical in fighting septic shock. Obvious problems should therefore be fixed, and they should be fixed as soon as possible. In this regard, it makes human (physiologic) sense that septic patients do benefit from early intervention and treatment. This assumption is supported by the fact that the awareness resulting from the Rivers et al. trial [14] led to a rapid change in practice. Since it is logical that time matters, early (goal-directed) treatment became a global standard. To which degree this concept contributed to the overall reduction in mortality rates of sepsis during the last decade remains unanswered, but it is most likely that it played a substantial role.

Avoidance and prevention of sepsis are undoubtedly the best route for the patient. However, if the "predator" is around you somewhere, be alert and prepared to act as fast and effectively as possible so that you have a chance to win the race against the "septic shark".

## Abbreviations

ACCP, American College of Chest Physicians; ARISE, Australian Resuscitation In Sepsis Evaluation; EGDT, Early goal-directed therapy; ICU, intensive care unit; MAP, mean arterial pressure; ProCESS, Protocolized Care for Early Septic Shock; ProMiSe, Protocolized Management in Sepsis; SCCM, Society of Critical Care Medicine; SIRS, Systemic inflammatory response syndrome; SIT, sepsis intervention team; SOFA, Sequential Organ Failure Assessment; SOS-D, salvage, optimization, stabilization, and de-escalation; SSC, Surviving Sepsis Campaign.

## Competing interests

MW is the Chief Medical Officer of Fresenius Kabi. The authors state that they have no competing interests concerning the submitted article.

## Notes

This article has been published as part of *Critical Care *Volume 19 Supplement 3, 2015: Future of Critical Care Medicine (FCCM) 2014. The full contents of the supplement are available online at http://www.ccforum.com/supplements/19/S3. Publication of this supplement was supported by Fresenius Kabi.
